# Reducing radiation exposure in second-generation cryoballoon ablation without compromising clinical outcome

**DOI:** 10.1007/s10840-020-00737-7

**Published:** 2020-04-13

**Authors:** Marijn J. Holl, Rohit E. Bhagwandien, Mehran Firouzi, Wouter A. de Ruiter, Tamas Szili-Torok, Sing-Chien Yap

**Affiliations:** 1 Department of Cardiology, Erasmus MC, University Medical Center Rotterdam, Rotterdam, the Netherlands; 2 Department of Cardiology, Maasstad Hospital, Rotterdam, the Netherlands; 3 Radiation Protection Unit, Erasmus MC, University Medical Center Rotterdam, Rotterdam, the Netherlands

**Keywords:** Atrial fibrillation, Cryoballoon ablation, Fluoroscopy, Pulmonary vein isolation, Radiation exposure

## Abstract

**Purpose:**

Pulmonary vein isolation (PVI) using cryoballoon (CB) ablation is associated with an increased radiation exposure compared with radiofrequency ablation. Previous studies showed that radiation exposure in CB PVI can be reduced by optimizing the fluoroscopy protocol without comprising acute efficacy and safety. We evaluated the mid-term outcome of a modified fluoroscopy protocol in patients undergoing CB PVI.

**Methods:**

The study population comprised 90 consecutive patients who underwent second-generation CB-based PVI. The first 46 patients underwent CB PVI with conventional fluoroscopy settings (group A, historic control group). In the following 44 patients (group B), a modified fluoroscopy protocol was applied consisting of (1) visualization of degree of PV occlusion only by fluoroscopy (no cine runs); (2) increased radiation awareness. Primary endpoints were the total dose area product (DAP), fluoroscopy time, and freedom from documented recurrence of atrial fibrillation (AF) after a single procedure.

**Results:**

Group B had a lower median DAP (1393 cGycm^2^ vs. 3232 cGycm^2^, *P* < 0.001) and median fluoroscopy time (20 min vs. 24 min, *P* < 0.001) as compared with group A. The 1-year freedom from documented recurrence of AF after a single procedure was similar among groups (74% in group A vs. 77% in group B, *P* = 0.71). There were no significant differences between both groups for the secondary endpoints, including procedure duration, proportion of patients with complete electrical isolation, and complications.

**Conclusion:**

Using a modified fluoroscopy protocol and increased radiation awareness, radiation exposure can be significantly reduced in CB PVI with a similar 1-year clinical outcome.

## Introduction

Pulmonary vein isolation (PVI) is the cornerstone of invasive treatment of symptomatic drug-refractory atrial fibrillation (AF). Currently, the two most frequently used ablation techniques for PVI are radiofrequency (RF) ablation followed by cryoballoon (CB) ablation. With RF ablation, continuous circumferential lesions are created around the pulmonary veins (PV) usually by point-by-point ablation. CB ablation offers a simpler and less operator-dependent means of achieving PVI by producing a large circular ablation zone. The landmark FIRE AND ICE trial and a recent meta-analysis showed that CB PVI was noninferior to RF ablation with respect to efficacy and safety [1, 2]. The CIRCA-DOSE trial reconfirmed the noninferiority of second-generation CB PVI when compared with contact-force-guided RF ablation [3]. Besides the less operator dependency, main advantages of CB ablation are the shorter procedure time and shorter left atrial (LA) dwell time [1]. The drawbacks of CB ablation are the increased risk of phrenic nerve palsy and increased radiation exposure in comparison to RF ablation [4]. CB PVI requires more extensive fluoroscopic guidance in comparison to RF ablation where catheter guidance can be achieved with 3D mapping. There is general consensus that radiation exposure should be kept as low as possible, not only for patients but also for catheterization laboratory staff [5]. Previous studies showed that radiation exposure in second-generation CB procedures can be reduced by optimizing the fluoroscopy protocol without compromising acute efficacy and safety [6, 7]. The aim of the present study is to evaluate the acute and mid-term outcome of a modified fluoroscopy protocol and increased radiation awareness in patients undergoing second-generation CB ablation.

## Methods

### Study population

The study population comprised consecutive patients who underwent their PVI using CB ablation from August 2016 to April 2018 in the Erasmus MC. In our institution, patients with AF are eligible for PVI using CB ablation when their PV anatomy is suitable based on their CT scan. Patients with a common ostium or a large PV (> 24 mm) were considered not suitable for CB ablation. Furthermore, patients requiring substrate ablation and/or a redo PVI were planned for RF ablation. The study population consisted of a historic control group (group A) using conventional fluoroscopy settings and an intervention group (group B) who had PVI using a modified fluoroscopy protocol (start date June 2017). The study was approved by the local Ethics Committee.

### Periprocedural management

All patients received oral anticoagulation for at least 4 weeks prior to ablation. Patients using vitamin K antagonist (VKA) underwent the procedure using uninterrupted anticoagulation with a target international normalized ratio (INR) between 2.0 and 3.0. Patients using non-vitamin-K oral anticoagulants (NOAC) skipped their dose on the morning of the procedure as previously described [8]. To exclude LA thrombi, all patients underwent transesophageal echocardiogram within 24 h of the procedure. After the procedure, patients using NOAC restarted their oral anticoagulation or low-molecular weight heparin 2 h after the procedure. The following day, an echocardiogram was performed to rule out pericardial effusion before discharge.

### Ablation procedure

In general, CB PVI was performed using conscious sedation. Based on patient preferences, procedures could be performed under general anesthesia. Vascular access was usually gained through the right femoral vein using an 8F and 10F sheath. A steerable decapolar catheter (Inquiry, Abbott, Lake Bluff, IL) was positioned in either the right ventricle for bradycardia pacing during freezing of the left PVs or in the vena cava superior for right phrenic nerve stimulation during freezing of the right PVs. Transseptal puncture was performed under fluoroscopic and intracardiac echocardiographic (ICE) guidance with the use of a SL1 transseptal sheath (Abbott, Lake Bluff, IL) and RF transseptal needle (Baylis Medical, Montreal, Canada). Heparin was given to maintain an activated clotting time of > 275 s, which was measured every 30 min. The transseptal sheath was exchanged over a guidewire for a 12F (outer diameter 15F) steerable transseptal sheath (FlexCath, Medtronic, Minneapolis, MN) through which a 28-mm CB (Arctic Front Advance, Medtronic, Minneapolis, MN) was advanced into the LA. No selective PV angiography was performed before balloon placement. A 20 mm spiral multipolar mapping catheter (Achieve, Medtronic, Minneapolis, MN) was used for guiding the CB to the target PV and real-time measurements of PV potentials during cryoablation. The degree of PV occlusion was measured by contrast injection through the central lumen of the inflated CB. The freezing time was depended on the time-to-isolation (TTI). A freezing cycle duration was set to 180 s when the TTI was < 60 s; otherwise, the freezing cycle duration was increased to 240 s. No additional bonus freeze cycle was applied after achieving PVI. If the PV was not isolated, additional freeze applications were performed with different alignment of the CB with respect to the target PV. If complete PVI could not be achieved, we did not switch to a RF catheter for a touch-up ablation. During CB ablation of the right PVs, the phrenic nerve function was monitored during pacing of the right phrenic nerve and manual palpation of the abdomen for diaphragmatic contractions. The freeze cycle was prematurely stopped after loss of diaphragmatic contractions during right PV ablation or when the cryoballoon temperature were below − 60 °C (probably too distal ablation site). Confirmation of durable PVI was performed 20 min after ablation of the corresponding PV.

### Fluoroscopy settings

**Fig. 1:**
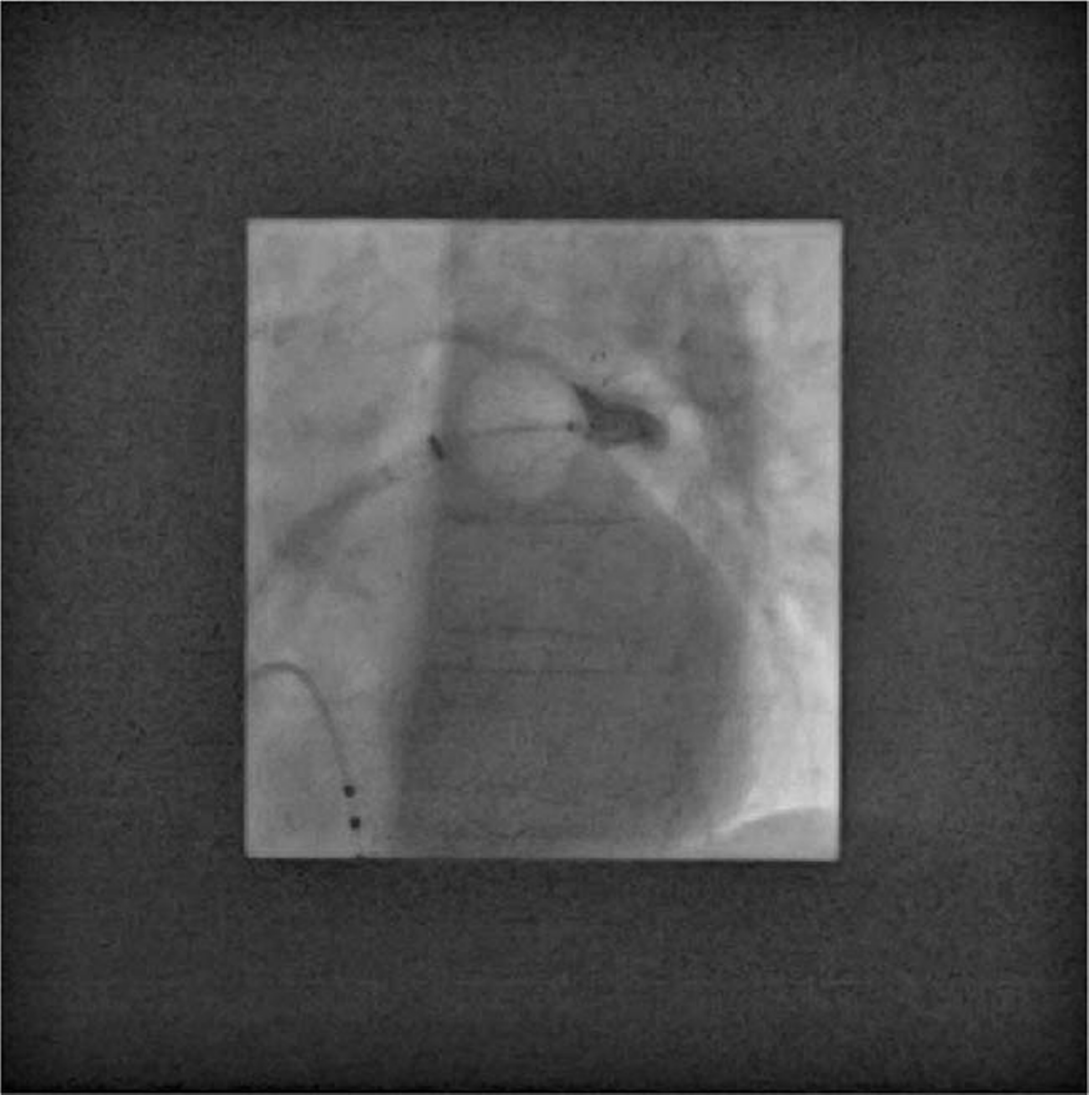
Example of stored fluoroscopic image during occlusion of the left superior pulmonary vein demonstrating the amount of collimation

Both groups underwent CB ablation using the same angiocardiography system (Allura FD20, Philips, the Netherlands) by a limited number of operators (REB, MF, SCY). The fluoroscopy frame rate in both groups was set to 7.5 pulses per second. During fluoroscopy, spectral filters containing 0.9 mm copper and 1.0 mm aluminum were used; cine runs were performed with filters containing 0.1 mm copper and 1.0 mm aluminum. In group A (conventional group), the degree of PV occlusion prior to each freeze cycle was filmed (cine run). In group B (intervention group), the following modifications were made: (1) degree of PV occlusion prior to each freeze cycle was only visualized by fluoroscopy and stored with the “store fluoroscopy” function (no cine run); (2) increased radiation awareness (e.g., optimal collimation, minimizing distance between patient and detector, limiting fluoroscopy) (Fig. 1).

### Follow-up

After the ablation procedure, outpatient clinic visits were routinely scheduled at 3, 6, and 12 months. These visits included a 12-lead electrocardiogram (ECG) and ambulatory 7-day Holter monitoring. Antiarrhythmic drug therapy was usually continued until the first outpatient clinic visit at 3 months after the ablation. If the patient was asymptomatic at the 3-month follow-up visit and no AF was documented, then usually antiarrhythmic medication was discontinued. If the patient had a low thromboembolic risk, as assessed using the CHA_2_DS_2_-VASc score (male < 1, female < 2), oral anticoagulation was stopped at the 3-month follow-up visit.

### Endpoints

The primary endpoints are the total dose area product (DAP); total fluoroscopy time; and freedom from documented recurrence of AF lasting longer than 30 s after a single procedure (after a blanking period of 3 months), with or without the use of antiarrhythmic drugs. Secondary endpoints are procedure duration, complete electrical isolation of PVs, and procedural complications.

### Statistical analysis

Continuous variables are presented as mean ± SD or median with corresponding 25th and 75th percentile, as appropriate. Categorical variables are presented by numbers and percentages. Difference of continuous variables between groups was analyzed with the unpaired Student *t* tests if the data were normally distributed, and with the Mann–Whitney *U* test otherwise. When comparing frequencies between groups, the chi-square or Fisher’s exact test was used, where appropriate. Cumulative freedom from AF recurrence was constructed with the use of the Kaplan-Meier method and groups were compared with log-rank statistics. Furthermore, logistic regression analysis was performed correcting for differences in baseline characteristics between groups. Data are presented as odds ratios and 95% confidence intervals (CI). Statistical analyses were performed using SPSS version 24 (IBM, Armonk, NY). All statistical tests were two-sided. A *P* value < 0.05 was considered statistically significant.

## Results

### Study population

A total of 90 patients underwent CB PVI within the study period. A total of 44 patients (group B) underwent the ablation using the modified fluoroscopy protocol starting from June 2017. Baseline characteristics of the study population are provided in Table 1. There was a higher proportion of females and smaller mean LA diameter in group B compared with group A. There were no differences in other baseline characteristics; especially, the type of AF was similar between both groups, with the majority of patients having paroxysmal AF.

**Table 1:** Patient characteristics

	Group A (*N* = 46)	Group B (*n* = 44)	*P* value
Age (years)	60 ± 10	57 ± 11	0.16
Female gender	7 (15%)	20 (46%)	0.002
BMI (kg/m^2^)	28 ± 3	27 ± 3	0.42
Obesity (BMI ≥ 30 kg/m^2^)	11 (24%)	9 (20%)	0.69
Hypertension	23 (50%)	16 (36%)	0.19
Diabetes mellitus	1 (2%)	2 (5%)	0.61
Prior stroke/TIA	2 (4%)	2 (5%)	1.00
CHA_2_DS_2_-VASc score			0.40
- 0	13 (28%)	14 (32%)	
- 1	13 (28%)	12 (27%)	
- 2	16 (35%)	13 (30%)	
- 3	–	3 (7%)	
- 4	4 (9%)	2 (5%)	
HAS-BLED score			0.43
- 0	26 (57%)	30 (68%)	
- 1	18 (39%)	12 (27%)	
- 2	1 (2%)	2 (5%)	
- 3	1 (2%)	–	
LA diameter (mm)	42 ± 5	38 ± 6	0.003
Ischemic cardiomyopathy	1 (2%)	0 (0%)	1.00
Type of AF			0.44
- Paroxysmal AF	41 (89%)	42 (96%)	
- Persistent AF	4 (9%)	2 (5%)	
- Long-standing persistent AF	1 (2%)	0 (0%)	
Presence of common ostium	1 (2%)	3 (7%)	0.36
General anesthesia	10 (22%)	11 (25%)	0.72

Continuous data are presented as mean ± SD. Categorical data are presented as *n* (%)

*AF* atrial fibrillation, *BMI* body mass index, *TIA* transient ischemic attack

### Primary endpoints

Procedural data are presented in Tables 2 and 3. Both the median total DAP (1393 [827, 2842] versus 3232 [2108, 5046] cGycm^2^, *P* < 0.001) and median fluoroscopy time (19 [12, 23] versus 24 [19, 30] min, *P* < 0.001) were lower in group B compared with group A. Even after correcting for the procedure time, the median indexed DAP (157 [95, 254] cGycm^2^/min versus 298 [233, 492] cGycm^2^/min, *P* < 0.001) and median indexed fluoroscopy time (0.19 [0.14, 0.22] versus 0.21 [0.19, 0.27], *P* = 0.005) remained lower in group B compared with group A.

**Table 2:** Ablation data

	Group A (*N* = 46)	Group B (*n* = 44)	*P* value
Total number of PVs	183	173	
Electrical isolation of all PVs	45 (98%)	39 (89%)	0.11
Left superior PV			
- Electrical isolation	44/45 (98%)	41/41 (100%)	1.00
- Number of applications			
- 1 application	35 (78%)	31 (76%)	0.81
- 180 s application	20 (44%)	17 (41%)	0.78
- 2 applications	5 (11%)	7 (17%)	
- > 2 applications	5 (11%)	3 (7%)	
- Minimal balloon temperature (°Celsius)	− 46 (− 51, − 43)	− 47 (− 51, − 44)	0.36
- Time to isolation (sec)*	55 (41, 77)	38 (32, 55)	0.13
Left inferior PV			
- Electrical isolation	44/45 (98%)	39/41 (95%)	0.60
- Number of applications:			
- 1 application	26 (58%)	25 (61%)	0.76
- 180 s application	16 (36%)	11 (27%)	0.38
- 2 applications	10 (22%)	10 (24%)	
- > 2 applications	9 (20%)	6 (15%)	
- Minimal balloon temperature (°Celsius)	− 42 (− 45, − 38)	− 42 (− 47, − 40)	0.34
- Time to isolation (sec)*	58 (33, 102)	86 (48, 104)	0.31
Right superior PV			
- Electrical isolation	46/46 (100%)	41/44 (93%)	0.11
- Number of applications:			
- 1 application	34 (74%)	32 (73%)	0.90
- 180 s application	25 (54%)	21 (48%)	0.53
- 2 applications	11 (24%)	9 (20%)	
- > 2 applications	1 (2%)	3 (7%)	
- Minimal balloon temperature (°Celsius)	− 48 (− 54, − 44)	− 52 (− 55, − 49)	0.08
- Time to isolation (sec)*	34 (28, 100)	45 (22, 60)	1.00
Right inferior PV			
- Electrical isolation	45/46 (98%)	42/44 (96%)	0.61
- Number of applications:			
- 1 application	34 (74%)	30 (68%)	0.55
- 180 s application	21 (46%)	14 (32%)	0.18
- 2 applications	7 (15%)	8 (18%)	
- > 2 applications	5 (11%)	6 (14%)	
- Minimal balloon temperature (°Celsius)	− 45 (− 52, − 40)	− 43 (− 48, − 39)	0.21
- Time to isolation (sec)*	45 (31, 61)	48 (37, 65)	0.66
Left common PV			
- Electrical isolation	1 (100%)	2/3 (67%)	1.00
- Number of applications			
- 1 application	–	1 (33%)	1.00
- > 2 applications	1 (100%)	2 (66%)	
- Minimal balloon temperature (°Celsius)	− 34	− 40 (− 41, − 39)	1.00

Continuous data are presented as median (IQR). Categorical data are presented as *n* (%)

*PV* pulmonary vein

*When available

**Table 3:** Fluoroscopy data

	Group A (*N* = 46)	Group B (*n* = 44)	*P* value
Total dose area product (cGycm^2^)	3232 (2108, 5046)	1393 (827, 2842)	< 0.001
Total effective dose (mSv)	6.5 (4.2, 10.1)	2.8 (1.7, 5.7)	< 0.001
Total fluoroscopy time (min)	24 (19, 30)	19 (12, 23)	< 0.001
Total dose (mGy)	194 (123, 279)	84 (55, 157)	< 0.001
Procedure duration (min)	108 ± 30	99 ± 22	0.13

Continuous data are presented as mean ± SD or median (IQR), as appropriate. Categorical data are presented as *n* (%)

Obesity (BMI ≥ 30 kg/m^2^) was associated with an almost twofold higher radiation dose within both cohorts (group A 5348 [3267, 8630] versus 2873 [2081, 4342] cGycm^2^, *P* = 0.03; group B 2401 [1934, 8322] versus 1262 [761, 2007] cGycm^2^, *P* = 0.003). When restricting the analysis to obese patients (*N* = 20), the radiation dose was similar between both groups (5348 [3267, 8630] versus 2401 [1934, 8322], *P* = 0.37, for groups A and B, respectively).

**Fig. 2:**
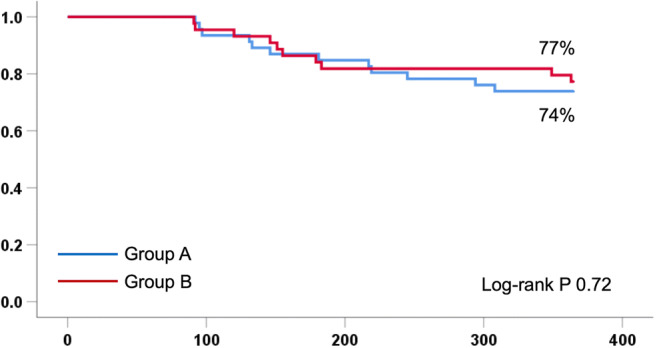
Freedom from AF with or without antiarrhythmic drugs stratified by group

The 1-year freedom from documented AF recurrence after a single procedure with or without antiarrhythmic drugs after a blanking period of 3 months was 74% in group A versus 77% in group B (*P* = 0.71) (Table 4, Fig. 2). The odds ratio for freedom of AF was 1.20 (95% CI, 0.46–3.15, *P* = 0.71) for group B in comparison with group A. The adjusted odds ratio after correcting for gender and LA dimension was 1.33 (95% CI, 0.45–3.93, *P* = 0.60).

**Table 4:** Efficacy outcome at 1 year

	Group A (*N* = 46)	Group B (*n* = 44)	*P* value
Freedom from AF, without antiarrhythmic drugs	33 (72%)	34 (77%)	0.55
Freedom from AF, with or without antiarrhythmic drugs	34 (74%)	34 (77%)	0.71
Freedom from atrial arrhythmia, without antiarrhythmic drugs	32 (70%)	33 (75%)	0.57
Freedom from atrial arrhythmia, with or without antiarrhythmic drugs	33 (72%)	33 (75%)	0.73
Second ablation procedure	7 (15%)	5 (11%)	0.59

Categorical data are presented as *n* (%)

*AF* atrial fibrillation

When the 1-year efficacy outcome was defined using different accepted definitions, the clinical outcome was still similar between groups (Table 4). Also, the 1-year rate of repeat ablations was similar between groups: 15% in group A and 11% in group B (*P* = 0.59).

### Secondary endpoints

The procedure duration and the proportion of patients with complete electrical isolation were similar between groups (Tables 2 and 3). Also, the rate of complications was similar; 3 patients in either group experienced a complication. In group A, one patient had a femoral pseudoaneurysm and two patients a phrenic nerve palsy. In group B, two patients had a vascular access-site bleeding and one patient suffered from a phrenic nerve palsy and pericarditis.

## Discussion

Using a modified fluoroscopy protocol and increased radiation awareness during second-generation CB-based PVI procedures, the radiation exposure could be reduced by more than 50% without compromising mid-term clinical outcome. The measures used in the present study to reduce radiation exposure can be easily implemented in any catheterization laboratory without a significant change in the approach to CB PVI.

### Advantages of cryoballoon ablation

PVI is the cornerstone of AF ablation and most patients with AF will receive only a PVI during their index ablation procedure. Previous studies have shown that the clinical efficacy and safety of PVI using either RF or CB ablation is comparable [1, 2, 9], independent of the use of contact-force catheters and the type of AF (paroxysmal versus non-paroxysmal) [3, 10–12]. During the past decade, CB ablation has emerged as an attractive single-shot alternative to RF ablation. This widespread adoption of CB ablation may be explained by its relative simplicity, lack of need of 3D mapping system, shorter procedure duration [1, 13], faster learning curve, and higher reproducibility (less operator-dependent) [14]. Disadvantages of the CB ablation are the technical challenges due to anatomical PV variants (i.e., common ostium, supernumerary veins) [15], higher risk of phrenic nerve palsy [1, 9], and higher radiation exposure [1, 4]. Radiation exposure can lead to acute and subacute skin injury, malignancy, and genetic abnormalities. Therefore, every attempt should be made to minimize radiation exposure according to the ALARA principle.

### Reducing radiation exposure

During a CB procedure, fluoroscopy is used for maneuvering of catheters and sheath, assisting with the transseptal puncture, and determining the degree of PV occlusion. Some operators also use selective PV angiography prior to balloon inflation to determine the PV and atrial anatomy. Various measures can be taken to reduce radiation exposure during CB ablation procedures [5]. Heightened awareness of the operator and team to reduce radiation exposure is the first step. Technical measures consist of lowering fluoroscopy and cine frame rates, limited use of cine, lowering of the detector on the patient, collimation of region of interest, and avoidance of left anterior oblique projections [6, 7]. Non-fluoroscopic measures are the use of ICE guidance, 3D CT overlay for PV anatomy, and real-time pressure waveforms to assess PV occlusion [7, 16–18]. Finally, using a novel angiography platform (e.g., Philips Azurion versus Allura system) can also reduce radiation exposure but this is not easily implemented [19].

Data on fluoroscopic measures to reduce radiation exposure in CB procedures and the effect on clinical outcome is limited [6, 7]. Rubesch-Kütemeyer et al. demonstrated that radiation exposure could be reduced by using ICE and optimized settings of the X-ray system in patients with paroxysmal AF [7]. ICE was used for evaluation of PV occlusion, as well as for guiding wires, mapping catheter, and the CB. Furthermore, PV angiography was skipped prior to CB inflation, fluoroscopy avoided whenever possible, frame rate reduced, distance between patient and detector minimized, and collimation was optimized. The mean DAP was reduced from 4935 ± 2094 cGycm^2^ to 1555 ± 1219 cGycm^2^ (*P* < 0.001). The 1-year freedom of AF was similar between groups (74% in the optimized group versus 78%, *P* = 0.64) [7]. Reissmann et al. demonstrated that by using an optimized fluoroscopy protocol without using ICE, a lower radiation exposure (median DAP, 389 versus 2168 cGycm^2^, *P* < 0.001) could be achieved during CB PVI in patients with paroxysmal or persistent AF [6]. The optimized fluoroscopy protocol in the latter study consisted of (1) avoidance of cine (store from fluoroscopy), (2) reducing the fluoroscopy frame rate (from 7.5 to 3.75 pulses per second) after the transseptal puncture, and (3) optimal collimation.

We demonstrate that by using a basic radiation reduction strategy, we could reduce the radiation exposure by more than 50% in comparison with the conventional approach. Our median effective dose in the optimized fluoroscopy group (2.8 mSv) was lower than typically encountered in an AF ablation procedure based on the literature (16.6 mSv) [4]. The reduction in radiation exposure remained after correcting for the procedure duration. This implies that reduction in radiation exposure was due to the implemented changes and not due to a more efficient or easier procedure secondary to increased operator experience, technical improvements, or different patient population. Furthermore, the 1-year freedom of AF was similar between groups (77% in the modified group versus 74% in the conventional group, *P* = 0.71). In contrast to the study of Rubesch-Kütemeyer et al. [7], we only used ICE for guidance of the transseptal puncture and not for guidance of catheters or evaluation of PV occlusion. Our modified fluoroscopy protocol was similar to the study of Reissmann et al. [5], but our study also provides data on mid-term outcome.

### Study limitations

There are certain limitations of the study. First, the observational nature of the study may introduce bias. The group with the modified fluoroscopy setting had a higher proportion of females and a smaller mean LA diameter. It is known that LA diameter is inversely correlated with clinical outcome after AF ablation; thus, this may positively influence the clinical outcome of the modified fluoroscopy group. However, even after correcting for sex and LA diameter, there was no difference in the 1-year freedom from documented AF between groups. Second, we did not systematically collect data on the number of PVs which showed small leaks during balloon occlusion between groups. Thus, we cannot comment on the differences in quality of balloon occlusion between groups. However, we did not require more balloon inflations in the group with the modified fluoroscopy protocol. Finally, this was a single-center study with a limited number of patients which may impact the generalizability of the results.

#### Conclusion

Radiation exposure during CB PVI can be reduced by using a modified fluoroscopy protocol and increased radiation awareness without compromising the acute and mid-term efficacy.
